# Transcriptional changes of aquaporin genes in leaves of black medic induced by arbuscular mycorrhizal fungal inoculation under water deficit

**DOI:** 10.18699/vjgb-26-46

**Published:** 2026-05

**Authors:** A.P. Yurkov, T.R. Kudriashova, A.I. Belyaeva, A.A. Kryukov

**Affiliations:** All-Russia Research Institute for Agricultural Microbiology, St. Petersburg, Russia; All-Russia Research Institute for Agricultural Microbiology, St. Petersburg, Russia; All-Russia Research Institute for Agricultural Microbiology, St. Petersburg, Russia; All-Russia Research Institute for Agricultural Microbiology, St. Petersburg, Russia

**Keywords:** aquaporins, AQP, arbuscular mycorrhiza, Medicago lupulina, Rhizophagus irregularis, drought, аквапорины, AQP, арбускулярная микориза, Medicago lupulina, Rhizophagus irregularis, засуха

## Abstract

One of the current research directions in plant-microbe interactions focuses on the mechanisms of plant adaptation to environmental stress through symbioses with various microorganisms. While the role of arbuscular mycorrhizal fungi in plant adaptation to drought is well-known, the underlying mechanisms of these processes remain poorly understood, particularly in leaf tissues. It is suggested that certain genes from the aquaporin family play a critical role both in adaptation to water deficit and in the development of an effective arbuscular mycorrhizal symbiosis. Thus, the important task in this study of plant-microbe symbioses is to assess the effect of arbuscular mycorrhizal fungal inoculation on the expression of aquaporin genes in leaves. This study utilizes the highly effective plant-microbe model system “Medicago lupulina + Rhizophagus irregularis” under drought stress conditions. A comparative assessment of gene transcription was carried out using the 2–ΔΔCT method based on real-time quantitative PCR results: normalization was performed relative to the actin reference gene with non-inoculated plants serving as the control. The study was conducted both at the initial development stage (the 2nd leaf stage), and at the stage of active plant-microbe interaction (the flowering stage). The study revealed genes with significant differential expression under drought conditions when comparing mycorrhizal and non-mycorrhizal Medicago lupulina plants: NIP3;1, NIP4;2, specific NIP7;1, TIP5;1 at the 2nd leaf stage; genes NIP3;1, NIP5;1, NIP6;4, NIP7;1 (specific), PIP1;4, TIP2;3 and specific XIP1;1 at the flowering stage. Previously, in a similar experiment, under well-watering conditions, the same genes did not have differential expression between mycorrhizal and non-mycorrhizal plants. Thus, the listed genes likely participate in the adaptation of the studied plants to drought conditions. The obtained information can be used to develop highly productive plant-microbe systems involving arbuscular mycorrhizal fungi, aimed at transitioning to organic farming, minimizing negative environmental impact, and enhancing plant resistance to water deficit

## Abstract (ru)

Одним из актуальных направлений исследований растительно-микробных взаимодействий является изучение механизмов адаптации растений к стресс-факторам среды за счет развития симбиозов с различными микроорганизмами. Хорошо известна роль грибов арбускулярной микоризы в адаптации растений к засухе, но механизмы данных процессов до сих пор раскрыты не полностью, особенно в тканях листьев. Предполагается, что некоторые из генов аквапоринов могут играть важную роль как в адаптации растений к недостатку влаги, так и в развитии эффективного симбиоза с грибами арбускулярной микоризы. Таким образом, важной задачей в изучении растительно-микробных симбиозов является оценка влияния инокуляции грибом арбускулярной микоризы на экспрессию генов аквапоринов в листьях растений в высокоэффективной модельной растительно-микробной системе “Medicago lupulina + Rhizophagus irregularis” в условиях засухи. Сравнительная оценка транскрипции генов выполнена методом 2–ΔΔCT по результатам количественной ПЦР в реальном времени: нормализация проведена по отношению к референсному гену – актину, контролем выступал вариант без инокуляции грибом. Исследование проведено как в начальную фазу развития – фазу развития 2-го листа, так и в фазу активного растительно-микробного взаимодействия – фазу цветения. В результате выявлены гены, имеющие достоверную дифференциальную экспрессию в условиях засухи при сравнении микоризованных и немикоризованных растений люцерны хмелевидной: NIP3;1, NIP4;2, специфический NIP7;1, TIP5;1 – в фазу развития 2-го листа; NIP3;1, NIP5;1, NIP6;4, NIP7;1 (специфический), PIP1;4, TIP2;3 и XIP1;1 (специфический) – в фазу цветения. Ранее в подобном эксперименте, но в условиях нормального полива эти же гены не имели дифференциальной экспрессии в сравнении микоризованных и немикоризованных растений. Вероятно, перечисленные гены принимают участие в адаптации изученных растений к условиям засухи. Полученные сведения могут быть использованы в разработке высокопродуктивных растительно-микробных систем с участием грибов арбускулярной микоризы с целью перехода к биологическому земледелию, минимизации негативного влияния на окружающую среду и повышению устойчивости растений к недостатку влаги

## Introduction

The problem of water deficit in crop production has become increasingly acute due to the disruption of natural ecosystems by intensive agriculture, excessive water withdrawal, and the weather conditions in various regions. To solve the problem of drought, a number of measures are employed, including not only irrigation, the introduction of new methods of no-till farming, shelterbelts, and crop rotation but also biological methods aimed at enhancing plant adaptation to environmental stress factors using symbiotic microorganisms. The role of arbuscular mycorrhiza (AM) fungi is well known; they likely played a key role in the colonization of land by plants approximately 400 million years ago (Remy et al., 1994). During that period, these fungi performed part of the functions of the root system, actively supplying plants with water and minerals. AM fungi support the ionic homeostasis of the host plant, provide osmotic protection and increase water use efficiency (Mammadov et al., 2018; Luo et al., 2022). Currently, more than 80 % of terrestrial plant species form AM with fungi from the class Glomeromycetes (Smith, Read, 2008). In this regard, a highly relevant area of research is to identify the mechanisms that control the adaptation of plants to soil moisture deficiency and the role of AM fungi in this process.

It should be assumed that plant adaptation to drought is closely related to the regulation of water transporters in plant tissues, among which the most represented group are aquaporins, small membrane proteins of the Major Intrinsic Proteins (MIP) family that form channels for transporting molecules across biological membranes (Maurel et al., 2015). In plants, they play a key role in adaptation to drought, salinity, and growth regulation (Daneliia et al., 2024; Kudriashova et al., 2025). The greatest diversity of aquaporins is characteristic of angiosperms, resulting from gene duplication via polyploidization (Singh et al., 2020), a process common to nearly all angiosperms. Polyploidization leads to gene duplication and the emergence of new isoforms. In particular, 35 aquaporin genes were found in Arabidopsis thaliana, 46 in Medicago truncatula, and 120 in rapeseed Brassica napus (Min et al., 2019; Daneliia et al., 2024). Aquaporins have a conservative structure. An important feature in their structure is that they form tetramers where each monomer functions as an independent channel (Kudoyarova et al., 2022). Aquaporin activity can be regulated by phosphorylation, pH, and redox reactions. Not all aquaporins are equally effective in water transport. Thus, aquaporins of angiosperms are divided into the five subfamilies: (1) NIP (nodulin 26-like intrinsic proteins) with low water permeability participate in the exchange of metabolites with microsymbionts (Kruse et al., 2006), and are localized in the plasma membrane and membranes of the endoplasmic reticulum (Ma et al., 2006; Mizutani et al., 2006; Lopez et al., 2016); (2) PIP (plasma membrane intrinsic proteins), permeable to water, hydrogen peroxide, and carbon dioxide, are localized in the plasma membrane, the inner membrane of chloroplasts, thylakoid, and the endoplasmic reticulum (Zhou et al., 2024); (3) SIP (small basic intrinsic proteins) with low permeability to water are localized on the membrane of the endoplasmic reticulum, and are poorly studied (Hussain et al., 2020; Zhou et al., 2024); (4) TIP (tonoplast intrinsic proteins), permeable to water, hydrogen peroxide, ammonium, and urea, are localized in the tonoplast (Maurel et al., 2008; Zhou et al., 2024); (5) XIPs (uncharacterized/X intrinsic proteins) with low water permeability are localized on the plasma membrane, and are poorly studied (Lopez et al., 2016; Noronha et al., 2016).

An analysis of the literature data suggests that the functions of aquaporins require significantly more investigation. Some studies indicate that mycorrhization increases plant resistance to drought. However, the mechanisms of aquaporin regulation remain unclear, especially regarding the species and tissue specificity. In particular, their expression in leaves is still under-researched (Daneliia et al., 2024; Kudriashova et al., 2025). Changes in aquaporin regulation mediated by mycorrhization remain an enigma (Sharma et al., 2021). Promising research directions include the identification of marker genes for effective symbioses (including AM symbiosis) that ensure plant adaptation to a lack of water in the substrate, the analysis of aquaporin gene expression under water stress, as well as the study of posttranslational modifications. From a practical perspective, such research will enable the development of symbiotically highly efficient and productive plant-microbial systems (PMS) necessary for the implementation of soilprotective resource-saving agriculture and, as a result, the production of environmentally friendly agricultural products.

Based on the above, the purpose of this study is to identify aquaporin genes in leaves that serve as markers of effective symbiosis. We employed a highly efficient model – PMS “Medicago lupulina + Rhizophagus irregularis” – under conditions of substrate moisture deficiency at the early and late stages of symbiosis development. For this purpose, the authors selected the highly responsive to mycorrhization line MlS-1 of Medicago lupulina. Previous transcriptomic analysis using the Massive Analysis of cDNA Ends (MACE-Seq) on this line identified over 4,500 differentially expressed genes ( padj < 0.01) in leaves upon colonization by the highly effective strain of the AM fungus Rhizophagus irregularis RCAM00320 (Yurkov et al., 2023).

## Materials and methods

Plant and fungal materials. Black medic (Medicago lupulina L.), which is a widespread species of the genus Medicago, diploid and self-pollinating, was used as a model plant. The MlS-1 line, which was bred from the VIK32 cultivar population and is characterized by its high sensitivity to mycorrhization, was selected as the object of the study (Yurkov et al., 2015). In the absence of arbuscular mycorrhizal (AM) fungal inoculation, plants of this line show signs of dwarfism under conditions of low plant-available phosphorus in the substrate (Yurkov et al., 2020). The RCAM00320 Rhizophagus irregularis strain from the ARRIAM collection, which has high symbiotic efficiency, was used for inoculation

Pot experiment. The experimental procedure was based on the protocol described by A.P. Yurkov et al. (2015). To prevent spontaneous infection by nodule bacteria and other microorganisms, the soil-sand mixture was sterilized (the substrate ratio was 2:1) by double autoclaving at 134 °C and 2 atm for 1 hour with an interval of 2 days. No substrate toxicity was detected after treatment of the mixture. Two Medicago seedlings were planted per pot, each containing 210 g of a soil-sand mixture. Half of the pots were inoculated with Plectranthus verticillatus roots colonized by R. irregularis, and in the other half (in the control), no inoculation was performed. Agrochemical parameters of the used soil were reported in the work of A.P. Yurkov et al. (2020): P2O5 content was 23 mg/kg and the pH was 6.44. The humidification conditions were selected based on a preliminary drought trial. The experimental design included variants with different moistening of plants, both with and without AM R. irregularis inoculation:

– “normal”, watering maintained at 0.6 of the total waterholding capacity (WHC); – “drought, variant 1”, transition from normal watering in the first 8 days to 0.4 of WHC for 16 days (until the 2nd leaf stage) and for 40 days (until the flowering stage); – “drought, variant 2”, transition from normal watering to 0.4 of WHC, for 7 days before harvesting at the 2nd leaf stage and the flowering stage (i. e., the drought regime was maintained for a plant for 7 days before harvest).

The third variant of drought (continuous 0.4 of WHC) was excluded due to high mortality rates in preliminary tests. In this preliminary experiment (Table S1)1, shoot fresh weight, symbiotic efficiency, plant mortality rate, and mycorrhizal infection frequency in the root were determined. Based on these indicators, drought variant 2 was selected for the analysis of the relative level of aquaporin gene expression in leaves.

Supplementary Materials are available in the online version of the paper: https://vavilov.elpub.ru/jour/manager/files/Suppl_Yurkov_Engl_30_3.pdf


In the main experiment, the first harvest was carried out 24 days after planting and inoculation, during the stage of the 2nd true leaf, and the second harvest – on day 48, during the flowering stage. Productivity parameters (shoot and root dry weight) and the effectiveness of symbiosis were evaluated according to previously described methods (Yurkov et al., 2015). The roots were stained with trypan blue (Phillips, Hayman, 1970). The calculation of mycorrhizal parameters, including the intensity of mycorrhizal infection in the root (M, %) and the abundance of arbuscules in the mycorrhizal part of the root (a, %), were carried out according to (Trouvelot et al., 1986) using specialized software (Vorobyev et al., 2016). To analyze gene expression levels, plant material was frozen in liquid nitrogen immediately after harvesting and stored at –80 °C until RNA extraction.

Isolation of RNA and analysis of gene expression. The selection of genes and primers for their amplification (Table S2) was carried out on the basis of M. truncatula data from the Phytozome genetic sequence database (https://phytozomenext. jgi.doe.gov/) and transcriptomic analysis of M. lupulina (Yurkov et al., 2023). The study included 30 genes from 46 known orthologs in M. truncatula, reported by K. Min et al. (2019). 16 genes had very low expression levels across all experimental variants, ranging from three to six orders of magnitude lower than the reference gene (with quantification cycles appearing more than 10 cycles later). Such low expression levels in the analysis are associated with a high error rate; therefore, these genes were excluded from further analysis. The absence of non-target PCR products was controlled by gel electrophoresis and analysis of melting curves. The efficiency of the primers was calculated using serial cDNA dilutions, primers with an efficiency close to 100 % (>95 %) were included in the work. The primers were tested at both sampling time points.

Total RNA from plant material was isolated using TRIzol reagent (Thermo Fisher Scientific, USA) with modifications (MacRae, 2007). Before cDNA synthesis, the quality of DNAase treatment was checked by PCR, cDNA synthesis was carried out from ~1 microgram of total RNA per sample using the Maxima First Strand cDNA Synthesis Kit with dsDNase according to manufacturer’s instructions (Thermo Fisher Scientific, USA). The quality of the obtained cDNA was checked by ubiquitin gene amplification. The level of gene expression was assessed by PCR-RT using a C1000 thermal cycler with a CFX-96 module (BioRad, USA) and a SYBR Green I reagent kit (Syntol, Russia). The amplification conditions were: initial denaturation – 95 °C, 5 min; 40 cycles – 95 °C, 15 s; 60 °C, 30 s; 72 °C, 30 s; followed by melt curve analysis. To compare the expression levels of the analyzed aquaporin genes, the 2–ΔΔCT method was used: the expression level in the variant with mycorrhization was compared against the control without AM. Normalization was performed relative to the reference gene, actin, according to (Yurkov et al., 2020). The PCR mix (volume – 10 μl) included: 1 μl of 10x B + SYBR Green buffer, 1 μl of 2.5 mM dNTP, 1 μl of MgCl2 (25 mM), 0.3 μl of each primer (10 mM), 0.125 μl (0.625 units) SynTaq DNA polymerase (Syntol, Russia), 4.275 μl of deionized water and 2 μl of cDNA. Each sample (both with and without AM) was analyzed in three biological and four technical replicates.

Statistical data processing was performed using ANOVA followed by Tukey’s post-hoc test with significance defined at p < 0.05. Student’s t-test (p <0.05) was used to compare the expression levels between the “+AM” and “–AM” groups.

## Results

The indicators of mycorrhization of M. lupulina and the effect of inoculation by the AM fungus on the growth, development of the host plant, and expression of aquaporin genes in leaves under drought conditions were evaluated. To simulate water stress, a drought regime was selected (based on a preliminary experiment; Table S1), consisting of watering at the rate of 0.4 of the total water-holding capacity (WHC) for one week prior to each experiment harvest, conducted 24 and 48 days post-inoculation.

**Fig. 1.**
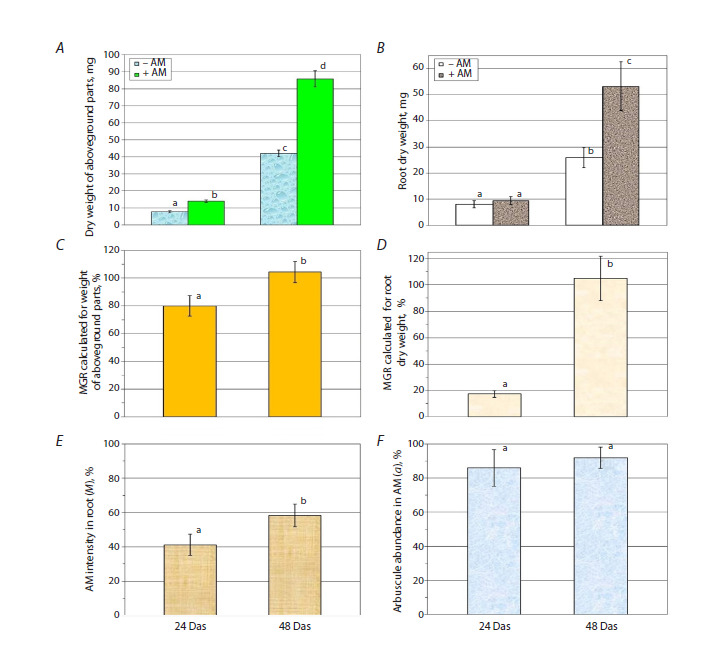
Indicators of productivity (A – shoot dry weight, mg, B – root dry weight, mg), symbiotic efficiency calculated by shoot weight, % (C) and root weight, % (D), and indicators of mycorrhization: E – intensity of mycorrhizal infection in the root (M); F – abundance of arbuscules in mycorrhizal part of the root (a) on days 24 and 48 after planting and inoculation. a, b, c – different letters above the columns indicate significant (p <0.05) differences.

M. lupulina productivity analysis (Fig. 1A, B) showed that plants inoculated with the fungus R. irregularis showed a significant increase in the shoot dry biomass at both the 2nd leaf stage (day 24) and the flowering stage (day 48). On the other hand, an increase in the root dry weight during mycorrhization was observed only at a late stage of development (at 48 days). Notably, the symbiotic efficiency of AM (MGR, Mycorrhizal Growth Response) was significant and high during the flowering stage (more than 100 %; Fig. 1C, D).

Microscopic analysis of M. lupulina roots showed the active development of mycorrhiza and arbuscules in the mycorrhized part of the root (Fig. 1E, F). There was an increase in the intensity of AM in the root (M) from 24 days to 48 days, and the abundance of arbuscules in the mycorrhized part of the root (a) was already high starting from the 2nd leaf stage; these results indicate a high activity of AM symbiosis under simulated drought conditions and provide a reliable basis for evaluating the effect of mycorrhization on the expression of aquaporin family genes in the leaves. Water stress was sufficient to induce mechanisms of adaptation to drought, but not critical for the survival of M. lupulina and maintaining a high level of mycorrhization.The indicators of AM efficiency and activity in conditions of lack of moisture (watering 0.4 MC of WHC) were comparable to the results obtained earlier with standard humidification (0.6 of WHC), in the model PMS “MlS-1 M. lupulina line + RCAM00320 R. irregularis strain” (Yurkov et al., 2021). This confirms that even under drought stress, it is possible to maintain a highly functional mycorrhizal symbiosis; furthermore, moderate drought stress stimulated a change in the transcription of aquaporin genes without suppressing the effectiveness of AM symbiosis.

**Fig. 2.**
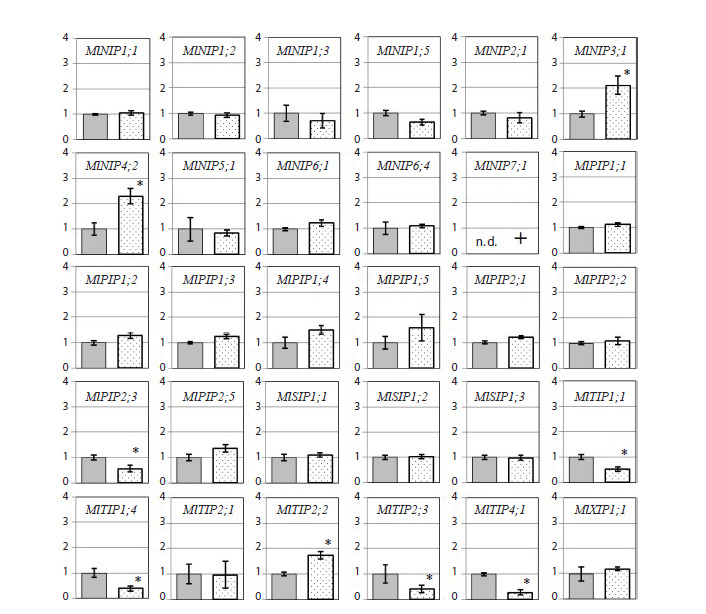
The relative level of transcripts (normalized value 2–ΔΔCT ) in M. lupulina leaves 24 days after sowing and inoculation with the AM fungus R. irregularis (during the 2nd leaf development stage) under drought condition. Here and in Figure 3: * Significant (p < 0.05) differences in the variant with and without AM. “+” the presence of specific expression in the variant. “n. d.” not detected.

**Fig. 3.**
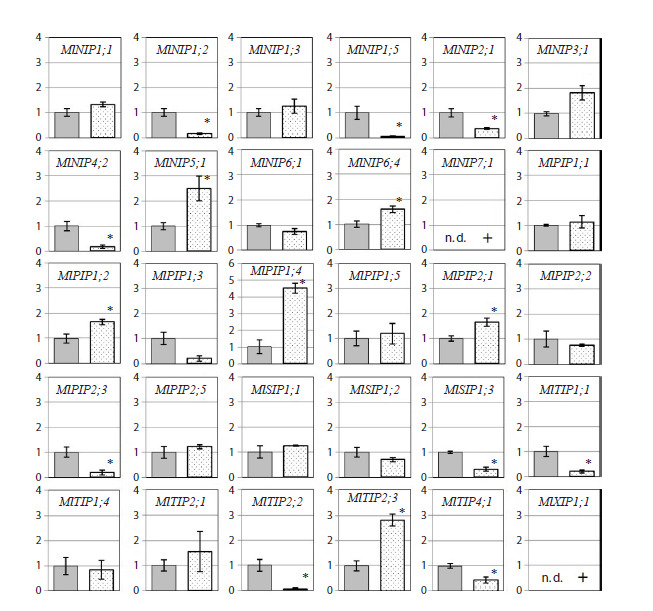
The relative level of transcripts (normalized value 2–ΔΔCT ) in M. lupulina leaves 48 days after sowing and inoculation with the AM fungus R. irregularis (during the flowering stage) under drought condition.

To address the study’s objectives, primers were selected and tested (Table S2). The analysis revealed that a subset of 30 aquaporin genes showed differential expression in M. lupulina leaves in response to mycorrhization under drought conditions (Fig. 2 and 3). Among these, genes with specific expression patterns were identified (marked with “+” for presence and “n. d.” for not detected, Fig. 2 and 3).

On day 24 (the stage of the 2nd true leaf), the following expression changes were observed in the leaves of mycorrhized plants (light columns) compared to the control (dark gray columns):– significant upregulation during mycorrhization (expression during mycorrhization was significantly (p < 0.05) higher than that in the control without inoculation by the AM fungus) for the NIP3;1, NIP4;2, TIP2;2 genes, as well as specific expression of NIP7;1; – significant downregulation during mycorrhization (expression during mycorrhization was significantly (p < 0.05) lower than that in the control without inoculation with the AM fungus) for the PIP2;3, TIP1;1, TIP1;4, TIP2;3, TIP4;1 genes.

On day 48 (the flowering stage), the following was detected in the leaves of plants inoculated with the AM fungus: – significant upregulation during mycorrhization of the NIP3;1, NIP5;1, NIP6;4, PIP1;2, PIP1;4, PIP2;1, TIP2;3 genes, as well as specific expression of NIP7;1, XIP1;1; – significant downregulation during mycorrhization of the NIP1;2, NIP1;5, NIP2;1, NIP4;2, PIP2;3, SIP1;3, TIP1;1, TIP2;2, TIP4;1 genes.

Thus, the most significant aquaporin genes involved in the development of effective AM symbiosis under water deficit have been identified. In particular, NIP7;1 demonstrated specific or induced expression during mycorrhization in both the vegetative and reproductive phases of the host plant. Special attention should also be paid to the XIP1;1 gene with specific expression during the flowering stage

The genes of the NIP subfamily generally exhibited upregulation (increased expression level (p < 0.05) relative to the level in the control without inoculation with AM fungus) in the early stage of AM symbiosis development, but by the late stage of development, the flowering stage, both up- and downregulation were observed for the genes of this family. This suggests complex regulation of aquaporins depending on the vegetative/reproductive stage of the host plant and with a restructuring of water metabolism under drought conditions during mycorrhization

## Discussion

The results indicate that aquaporins of the NIP and TIP subfamilies are actively involved in the development of AM symbiosis under drought conditions. However, according to research, only TIP aquaporins significantly affect the transmembrane water transport (Zhou et al., 2024). At the same time, NIP aquaporins transport diverse substrates, including metalloids, but have low water permeability. It is assumed that NIP proteins are involved in the exchange of metabolites between the host plant and symbiotic microorganisms (Kruse et al., 2006). It is also known that plants may downregulate aquaporin gene expression under drought to conserve water; therefore, special attention should be paid to genes, the expression of which is suppressed under conditions of water deficit (Quiroga et al., 2019). At the same time, according to other data, water deficit can trigger the activation of certain aquaporin genes that play a key role in plant resistance to drought (Jia, Liu, 2020; Zhou et al., 2024). Thus, the relationship between the expression of these genes, the effectiveness of plant symbiosis with AM fungi, and drought resistance remains poorly understood (Sharma et al., 2021). Further investigation could elucidate the mechanisms of adaptation of AM plants to water stress

The results of this study indicate that the selected irrigation regime (0.4 of WHC for 7 days prior to harvest) was sufficient for the development of symbiosis, as evidenced by the significant response to mycorrhization and the high frequency of AM fungal colonization in the roots of the host plant (refer to “drought, variant 2”, Table S1). Meanwhile, productivity indicators were reduced compared to those under standard watering conditions at 0.6 of WHC (Yurkov et al., 2020). It was shown that the symbiotic efficiency of the model system “M. lupulina + R. irregularis” under water deficit (0.4 of WHC) was high at both the early and late developmental stages. At 24 days (2nd leaf stage), a significant increase in shoot biomass due to mycorrhization was observed. At 48 days (flowering stage), the system was characterized by higher symbiotic efficiency in terms of both shoots and roots dry weight, as well as a higher activity of AM development, reflected in increased root mycorrhization intensity (M, %). Meanwhile, the abundance of arbuscules in the mycorrhizal part of the root (a, %) was already high at the early stage of symbiosis development

It was previously shown that under well-watered conditions (0.6 of WHC) (Yurkov et al., 2021), the indicators of AM efficiency and activity were also high. Thus, the selected drought regime caused stress sufficient to change the expression of a number of genes, but not critical for the functioning of the AM symbiosis itself. However, the role of aquaporins in the development of effective PMS under water deficit, as well as their regulation during mycorrhiza in general, has not been sufficiently studied. This is due to the broad substrate specificity of these transporters, and their diverse functions depending on the type of tissue and subcellular localization (Daneliia et al., 2024; Kudriashova et al., 2025). The data obtained in this study revealed multidirectional changes in gene expression depending on the subfamily and phase of development: in the early phase (24 days), upregulation of NIP subfamily genes (NIP3;1, NIP4;2, and specific expression of NIP7;1) was observed against the background of general downregulation of TIP subfamily genes (TIP1;1, TIP1;4, TIP2;3, TIP4;1). At the late stage (48 days), the parity between up- and downregulation was found in the NIP subfamily (four genes each, including one gene with specific expression during mycorrhiza – NIP7;1). The TIP subfamily, as in the early phase, was characterized by the predominance of downregulation (three genes). Furthermore, a gene from the XIP subfamily (XIP1;1) with specific expression upon mycorrhization during the flowering stage was also identified. It should be noted that the XIP subfamily remains poorly understood, though. XIP proteins are known to be localized on the plasma membrane (Lopez et al., 2016; Noronha et al., 2016).

It is of particular interest to compare the data obtained in this study under drought conditions (at 0.4 of WHC) with the transcriptomic analysis of mycorrhizal and non-mycorrhizal M. lupulina plants under normal watering conditions (at 0.6 of WHC; Yurkov et al., 2023). Only one gene, TIP2;2 (one of four upregulated genes under drought conditions), also had upregulation under normal watering conditions during the 2nd leaf stage, as well as PIP1;2 and PIP2;1 (two of eight) during the flowering stage (Yurkov et al., 2023). Among the genes characterized by a decrease in expression under drought, only the NIP1;5 gene (one out of nine) had a similar reduction in transcription under normal watering (Yurkov et al., 2023). Thus, the remaining genes that exhibited differential expression under drought conditions in the current study, but showed no such response under normal watering conditions in the 2023 experiment, may be considered candidate genes for the adaptation of M. lupulina plants to drought conditions. This list includes: NIP3;1, NIP4;2, specific NIP7;1, TIP5;1 – at the 2nd leaf stage; NIP3;1, NIP5;1, NIP6;4, NIP7;1 (specific), PIP1;4, TIP2;3 and XIP1;1 (specific) – at the flowering stage.

A comparative analysis with other PMS revealed several parallels (Asadollahi et al., 2023; Daneliia et al., 2024; Wang et al., 2024; Kudriashova et al., 2025). In the PMS “Zea mays + R. irregularis”, the ZmTIP2;3 gene was also upregulated during mycorrhization and presumably played an important role in increasing the resistance of corn to drought stress. However, this PMS was characterized by a lower responsiveness to inoculation by the AM fungus, since only a few maize genes (ZmPIP1;6, ZmPIP2;2, ZmTIP2;3, ZmTIP4;1) exhibited significant expression changes in response to mycorrhization. On the other hand, downregulation was observed in maize leaves during mycorrhization at the development stages of the 2nd and 13th leaves for some TIP subfamily genes (ZmTIP3;1, ZmTIP4;4 – in both phases; ZmTIP4;3 – only in 13th leaf stage) (Wang et al., 2024). The downregulation of certain TIP subfamily genes (PtTIP2;1 and PtTIP 5;1) under drought conditions was also shown in the roots of the PMS “Poncirus trifoliata + F. mosseae” (He J.-D. et al., 2019). So, while the results for PMS “Z. mays + R. irregularis” (on day 56) are consistent with our results for PMS “M. lupulina + R. irregularis” (on day 48) for the TIP2;3 gene, it is necessary to note the phase-dependent nature of the effects (dependence on the stage of plant development), as well as species- and tissue-specific patterns in aquaporin expression

Supporting this high tissue specificity, previous assessment of expression in the roots of the PMS “M. lupulina + R. irregularis” (Kryukov et al., 2025) showed that during the vegetative stage (2nd leaf stage), mycorrhization led to the upregulation of the NIP1;2, NIP1;5, TIP2;1 genes, the specific expression of NIP4;2, and the downregulation of NIP2;1, NIP3;1, PIP1;4, TIP3;1, XIP1;1. At the same time, during the generative phase (flowering), only two genes with specific expression were identified (NIP4;1, NIP7;1), whereas a large group of 14 genes across the NIP, PIP, SIP, TIP, and XIP subfamilies was downregulated (Kryukov et al., 2025). Thus, a comparative analysis revealed tissue-specific expression of the aquaporin family genes: only the NIP4;2 gene had upregulation in both roots and leaves in the PMS “M. lupulina + R. irregularis” in the vegetative stage; and only the NIP7;1 gene had specific expression during mycorrhization in the generative phase. However, a spectrum of genes with downregulation in both leaves and roots during mycorrhization in the flowering stage was broader – NIP2;1, SIP1;3, TIP2;2, TIP4;1. The phase-dependent effect of marker genes for the development of effective AM symbiosis has also been identified in other gene families, for example, in the SWEET sugar transporters (Kryukov et al., 2023; Kudryashova et al., 2024; Kudriashova et al., 2025). Notably, TIP2;3 was the only gene in the TIP subfamily that switched from downregulation to upregulation between the early (24 days) and the late (48 days) stages of mycorrhization. It is possible that TIP2;2 functionally compensates at the stage of the 2nd leaf, since the latter showed a reverse switch from up- to downregulation. Similar hypotheses about the compensatory mechanism of transcription of other aquaporins have been proposed by R. Porcel et al. (Porcel et al., 2005; Sharma et al., 2021). The tissue-specific expression of TIP and PIP aquaporins is often linked to increased water exchange and subsequent plant growth stimulation (He F. et al., 2016). For example, the expression levels of RpTIP2;1 and RpPIP2;1 consistently increased in different tissues – leaves, stem, and roots under mycorrhization and drought, directing water flows toward plant tissues, whereas RpTIP1;3 and RpPIP1;3 were downregulated in the leaves of the PMS “Robinia pseudoacacia + R. irregularis” (He F. et al., 2016).

In the present study, downregulation of the PIP subfamily (PIP2;3 gene) was shown in the PMS “M. lupulina + R. irregularis” both at the early (2nd leaf stage) and late (flowering) stages of development. Interestingly, according to literature data, higher activity of PIP proteins should enhance water conductivity under drought conditions. However, this hypothesis was challenged by experiments in the PMS “Glycine max + F. mosseae” and “Lactuca sativa + F. mosseae”: in those studies, downregulation of PIP genes (GmPIP1, GmPIP2, LsPIP1, LsPIP2) was observed (Porcel et al., 2006). This AM-mediated effect represents a regulatory mechanism that allows the host plant to conserve metabolic resources and minimize water loss under stress conditions. According to J.M. Ruiz-Lozano and R. Aroca (2010), the AM fungi help the plant retain absorbed water by reducing membrane permeability through the suppression of PIP aquaporin expression. On the other hand, since the hyphae of the fungus can supply the host plant with water themselves, this may be a strategy that allows AM plants to depend less on the intensive expression of their own aquaporins and thus save energy (Sharma et al., 2021). Based on the functional studies of aquaporins, it can be assumed that the TIP subfamily includes the main water transporters through the vacuolar membrane (tonoplast) (Zhou et al., 2024). Their suppression at the late stages of symbiosis likely contributes to minimizing water loss and optimizing cellular homeostasis (Quiroga et al., 2019). In other words, the downregulation of TIP genes at the flowering stage may reflect the strategy of water conservation in the PMS “M. lupulina + R. irregularis”.

Aquaporins of the NIP subfamily are involved in the transport of various substrates, including toxic metalloids and plant glycerol (Dean et al., 1999). They play an important role in the development of interaction between host plant and symbiotic microorganisms (Kruse et al., 2006). Aquaporins research began with the study of the NLM protein from the NIP subfamily, discovered in the peribacteroid membrane of symbiotic soybean root nodules (Sandal, Marcker, 1988). Aquaporins of the NIP subfamily are also found in non-leguminous plants (Kruse et al., 2006). Thus, it can be assumed that aquaporins of the NIP subfamily play an active role in the formation and development of effective symbiosis with the AM fungus in the PMS “M. lupulina + R. irregularis”. It is known that NIP aquaporins are closer in structure to bacterial AqpZ than to glycerol transporters from the GLP subfamily (Heymann, Engel, 1999). This indicates that the ancestral plant aquaporin probably did not have the ability to transport glycerol, and this function arose later in the evolution as compensation for the lack of GLP in plants (Murata et al., 2000). During adaptation to drought, the transport of glycerol in the leaves of the host plant in the PMS “M. lupulina + R. irregularis” may play an important role.

The upregulation of NIP3;1, NIP4;2 as well as the specific expression of NIP7;1 suggest that they play a role in the early stages of the symbiosis of “M. lupulina + R. irregularis”. The parity between up- and downregulation in the NIP subfamily in the leaves at the late stage of the symbiosis “M. lupulina + R. irregularis” indicates a phase-dependent effect of mycorrhization on the expression of aquaporins. At the same time, aquaporin genes – NIP3;1 and the specific NIP7;1 – maintained upregulation across the change of developmental stages. Thus, mycorrhization by the AM fungus R. irregularis modulates the expression of aquaporins in a phase-specific manner. We propose that the NIP3;1 and specific NIP7;1 may serve as positive markers for effective AM development under drought. This activation of NIP is probably related to the active metabolic exchange in AM symbiosis. The proportion of downregulated aquaporins under drought during mycorrhization may depend on the duration of stress. For example, in the experiments of G. Bárzana et al. (2014), in the PMS “Zea mays + R. intradices”, the downregulation of only ZmNIP2;1, and ZmNIP2;2 was observed during short-term drought (4 days), whereas extended drought (up to 12 days) led to the suppression of a significant part of aquaporin genes (ZmPIP1;1, ZmPIP1;2, ZmPIP1;3, ZmPIP1;4, ZmPIP2;2, ZmPIP2;4, ZmNIP2;1, ZmNIP2;2, ZmTIP1;1, ZmTIP1;2). A subsequent increase in the duration of drought (up to 42 days) in the PMS “Poncirus trifoliata + F. mosseae” resulted in the suppression of all studied aquaporin genes in the roots (Zou et al., 2019). Variations in the expression of the same isoforms of aquaporin may stem not only from the type of plant tissue, but also from the specific features of plant interaction with different AM fungal species (Bárzana et al., 2014; Sharma et al., 2021).

On the other hand, it is possible to assume the existence of other mechanisms for drought resistance in mycorrhizal plants due to the upregulation of the PIP and TIP subfamilies (in this study, upregulation of PIP1;2, PIP1;4, PIP2;1, and TIP2;3 in the flowering stage was observed). So, according to K. Sharma et al. (2021), the increased activity of PIP and TIP proteins is directly related to the resistance of plants to drought through several pathways: (1) increasing the efficiency of water transport (root hydraulic conductivity); (2) intensification of nutrient metabolism, in particular, nitrogen absorption; (3) enhancing photosynthesis (by the participation of aquaporins in CO2 diffusion) and increased production of photosynthetic substances (Uehlein et al., 2003; Bárzana et al., 2012); (4) maintaining osmotic pressure and turgor, including through regulation of stomata, improving the aquatic status of plants by increasing water use efficiency (Sharma et al., 2021). It is noteworthy that there are few studies elucidating the reasons for the downregulation of aquaporins under drought in mycorrhizal conditions (Asadollahi et al., 2023), which underlines the relevance of our analysis.

Thus we demonstrate that mycorrhizal symbiosis significantly influenced the water status of the host plant in the PMS “M. lupulina + R. irregularis” by modulating aquaporins expression under drought conditions

## Conclusion

This study showed that, despite the water deficit conditions, the plant-microbial system “M. lupulina + R. irregularis” demonstrated high rates of mycorrhization and symbiotic efficiency. In the early stage (day 24, 2nd leaf), a multi-directional regulation was observed: activation of the NIP3;1, NIP4;2, TIP2;2 genes, specific expression of NIP7;1, and suppression of other genes (PIP2;3, TIP1;1, TIP1;4, TIP2;3, TIP4;1). The SIP subfamily showed no significant changes. In the late phase (day 48, flowering), a more pronounced downregulation of the expression of most genes was revealed (NIP1;2, NIP1;5, NIP2;1, NIP4;2, PIP2; 3, SIP1;3, TIP1;1, TIP2;2, TIP4;1). The upregulation among the main water transporters was maintained for PIP1;2, PIP1;4, PIP2;1, TIP2;3. Two genes (NIP7;1, XIP1;1) remained active, which may indicate their specific role in the symbiosis. However, the TIP2;2, PIP1;2, PIP2;1 and NIP1;5 genes cannot be markers for effective AM development under drought due to the fact that they have similar regulatory patterns under well-watered conditions based on a comparative analysis with previously published transcriptomic profiles of M. lupulina leaves.

Functionally, the TIP subfamily genes, which encoded proteins that regulate water transport across the vacuolar membrane, showed the most significant downregulation, both at an early and late stages. They are probably involved in osmoregulation during water stress. The NIP genes are presumably involved in the processes of interaction with the symbiotic fungus, as evidenced by their selective activation. The role of the genes of the XIP subfamily in the response to drought during mycorrhization has yet to be studied. Our findings expand the current understanding of the mechanisms of adaptation of mycorrhizal plants to drought, in particular: (1) confirm the key role of aquaporins of the TIP subfamily in the regulation of water balance; (2) indicate the possible specificity of NIP genes for mycorrhizal symbiosis; (3) demonstrate the phase-dependent nature of regulation of aquaporin expression, which emphasizes the complexity of their interaction with the symbiont fungus. Thus, the study contributes to the understanding of the molecular basis of drought tolerance in mycorrhizal plants and opens new perspectives for further analysis of the role of individual aquaporin genes in symbiotic systems.

## Conflict of interest

The authors declare no conflict of interest.
